# A Novel *PCK1* Gene Variant Associated With Cytosolic Phosphoenolpyruvate Carboxykinase Deficiency: Two Siblings With Different Clinical Presentations

**DOI:** 10.1002/jmd2.70065

**Published:** 2026-01-26

**Authors:** Lauma Vasiļevska, Ieva Puķīte, Madara Auzenbaha, Dmitrijs Rots, Ieva Grīnfelde, Andžela Lazdāne, Sebastian Schulz‐Jürgensen

**Affiliations:** ^1^ Clinic of Medical Genetics and Prenatal Diagnostics Children's Clinical University Hospital Riga Latvia; ^2^ Department of Pediatric Gastroenterology Children's Clinical University Hospital Riga Latvia; ^3^ Riga Stradiņš University Riga Latvia; ^4^ Genetics Laboratory Children's Clinical University Hospital Riga Latvia; ^5^ Department of Pediatrics University Medical Center Eppendorf Hamburg Germany

**Keywords:** acute liver failure, PCK1 gene, phosphoenolpyruvate carboxykinase deficiency

## Abstract

Cytosolic phosphoenolpyruvate carboxykinase (PEPCK‐C) deficiency is a rare autosomal recessive gluconeogenesis disorder caused by variants in the *PCK1* gene. Clinically, PEPCK‐C deficiency is characterized by recurrent episodes of fasting‐induced hypoglycemia, liver dysfunction, and seizures, with the first hypoglycemic episode typically occurring in the neonatal period or in early childhood. We report a case of PEPCK‐C deficiency in an 8‐year‐old who presented with transient severe acute liver and kidney failure, accompanied by markedly elevated glutamine levels as the initial manifestation of the disease. The acute liver failure was reversible following continuous glucose infusion. Next‐generation sequencing identified two variants in the *PCK1* gene: one previously known pathogenic variant, c.925G>A p.(Gly309Arg), and a second previously unreported variant, c.1833_1834del p.(Glu611AspfsTer16). These variants were confirmed to be in a compound heterozygous state. Based on the patient's clinical presentation, the second variant was classified as likely pathogenic. Subsequent genetic testing of family members revealed that the patient's 12‐year‐old sister has the same *PCK1* variants but remains asymptomatic to date. Given the clinical findings, we propose that the c.1833_1834del p.(Glu611AspfsTer16) variant in the *PCK1* gene should be classified as likely pathogenic. We recommend considering molecular diagnostics for PEPCK‐C deficiency in patients presenting with severe acute liver failure and elevated glutamine levels, as early diagnosis and intervention may lead to a reversible outcome.

## Key Points/Highlights

- PEPCK‐C deficiency may present as reversible severe acute liver failure with hyperglutaminemia.
- A novel PCK1 frameshift variant, c.1833_1834del, was identified and classified as likely pathogenic.
- Early glucose infusion can reverse acute liver failure in PEPCK‐C deficiency.
- Molecular testing should be considered in unexplained acute liver failure with elevated glutamine.

## 1 Introduction

Cytosolic phosphoenolpyruvate carboxykinase or PEPCK‐C (EC: 4.1.1.32) deficiency (OMIM #261680) is a rare autosomal recessive gluconeogenesis disorder [1]. Clinically, PEPCK‐C deficiency is characterized by recurrent fasting‐associated hypoglycemia, liver dysfunction and seizures and biochemically, by lactic acidosis and excessive urinary excretion of tricarboxylic acid (TCA) cycle metabolites [2, 3]. Individuals with PEPCK‐C deficiency experience a wide range of clinical symptoms and severity levels. These can range from minimal liver involvement, normal development and hypoglycemia to a severe and fatal disease characterized by liver failure, hyperammonemia, and encephalopathy [4].

This disorder is caused by defects in the *PCK1* gene, which is the main control point for the regulation of gluconeogenesis and encodes cytosolic phosphoenolpyruvate carboxykinase‐1 [5]. A precise diagnosis can be established via molecular analysis. The most common pathogenic variant, c.925G>A p.(Gly309Arg), has been identified in 84% of patients and appears to be common in the Finnish population [2].

In this article, we report two sisters of Latvian origin. The 8‐year‐old sister developed acute transitory liver and kidney failure as her first PEPCK‐C presentation, whereas the 12‐year‐old sister remained asymptomatic. None of them had hypoglycemic episodes in the past. Both are compound heterozygous for a known common variant and an unreported *PCK1* variant identified by next‐generation sequencing.

## 2 Case Report

An 8‐year‐old girl was admitted to the emergency department with symptoms of drowsiness, jaundice and reduced urine output. In the 5 days prior to admission, she had experienced vomiting and low‐grade fever. Her symptoms began after consuming chocolate drink mixed with 24 sugar packets—approximately 72 g (g) of sucrose. On examination, she appeared somnolent and had jaundice of the skin and sclera. Abdominal examination revealed tenderness on the right side and an enlarged liver extending 6 cm below the right costal margin. Her weight was 27 kg (28th percentile), and her height was 134 cm (54th percentile).

She was born at term from unrelated parents and experienced prolonged neonatal jaundice. Her growth and development have been normal, with no chronic health issues noted. She had never been hospitalized, aside from a single emergency department visit for laryngitis at age 2. There is no history of food intolerance.

The laboratory tests showed severe liver dysfunction and acute kidney injury accompanied by oliguria with a glomerular filtration rate of 20 mL/min/1.73 m^2^ (calculated using the Schwartz equation). Additional findings included hypoketotic hypoglycemia, hyponatremia, hypochloremia, and coagulopathy (see Table 1).

**TABLE 1:** Laboratory findings at initial presentation.

Test	Result	Reference range	Test	Result	Reference range
ALT	6759 ↑	0–33 U/L	AST	9165 ↑	0–35 U/L
Bilirubin (Total)	99 ↑	0–17 μmol/L	Bilirubin (Direct)	92 ↑	0–5 μmol/L
GGT	86 ↑	0–17 U/L	Albumin	36 ↓	38–54 g/L
Creatinine	250 ↑	35–53 μmol/L	Urea	37.92 ↑	1.79–6.43 mmol/L
Na	123.5 ↓	136–145 mmol/L	K	4.21	3.5–5.1 mmol/L
Cl	86.91 ↓	98–107 mmol/L	pH (venous)	7.345 ↓	7.37–7.45
Prothrombin	30.6 ↓	86%–116%	INR	2.40 ↑	0.8–1.2
APTT	39.4	24–43 s	Fibrinogen	1.73 ↓	2.08–3.98 g/L
D‐dimers	4.11 ↑	0–0.55 mg/L	CRP	10 ↑	0–5 mg/L
LDH	2151 ↑	120–300 U/L	Lactate	2.79 ↑	0.6–0.9 mmol/L
Glucose	1.56 ↓	3.33–5.55 mmol/L	Ammonium	56.53 ↑	11–51 μmol/L

Abbreviations: ALT, alanine transaminase; APTT, activated partial thromboplastin time; AST, aspartate transaminase; CRP, C‐reactive protein; GGT, gamma‐glutamyl‐transferase; INR, international normalized ratio; LDH, lactate dehydrogenase.

She fulfilled all the diagnostic criteria for pediatric acute liver failure (PALF): acute onset of clinical liver disease without prior evidence of chronic liver pathology, biochemical evidence of acute liver injury (AST and ALT > 100 U/L), biliary dysfunction and coagulopathy with an INR > 2.0 that remained elevated despite vitamin K administration [6].

Laboratory results were negative for hepatitis A, hepatitis B, and hepatitis C, human immunodeficiency virus, and cytomegalovirus. Stool analysis by reverse transcription–polymerase chain reaction (RT‐PCR) was positive for enteropathogenic *E. coli* . Serological testing indicated a latent Epstein–Barr virus (EBV) infection, with no detectable EBV DNA in the serum. Abdominal ultrasound showed hepatomegaly (liver enlarged by 6 cm below the rib cage), increased hepatic echogenicity, edema of both the liver and kidney parenchyma, and mesenteric lymphadenopathy. Plasma and urine amino acid analysis revealed elevated levels of nearly all amino acids (see Table 2).

**TABLE 2:** Amino acid profile in plasma and urine.

Amino acid	Plasma, μmol/L	Plasma reference interval, μmol/L	Urine, mmol/mol creatinine	Urine reference interval, mmol/mol creatinine
Alanine	748.7 ↑	158–314	241.9 ↑	8–80
Arginine	104.5 ↑	38–98	5.4	0–9
Citrulline	25.3	18–50	2.3 ↓	4–18
Glutamine	3217.1 ↑	373–709	2903.5 ↑	7–137
Glycine	769.1 ↑	113–261	922.7 ↑	20–201
Lysine	260.3 ↑	77–181	71.9 ↑	0–64
Methionine	59.4 ↑	11–27	13.4 ↑	0–8
Ornithine	147.9 ↑	24–64	12.7 ↑	0–7
Phenylalanine	81.5 ↑	35–67	27.9 ↑	1–20
Proline	569.1 ↑	93–221	33.2 ↑	0–7
Serine	214.9 ↑	77–169	390.8 ↑	1–95
Threonine	225.7 ↑	48–140	401.2 ↑	2–45
Tyrosine	35.8	34–82	26.7	6–42

Markedly elevated glutamine levels were found in both plasma and urine, despite normal ammonium concentration. Organic acid analysis revealed hydroxy‐dicarboxylic aciduria, with increased excretion of 3‐hydroxyadipic lactone, 2‐hydroxyadipic acid, 3‐hydroxyadipic acid, and 3‐hydroxysebacic acid. Among the dicarboxylic acids, only adipic acid showed a significant increase. No elevation in ketone bodies was detected. Elevated levels of TCA cycle intermediates—inclusive of fumarate, malate, and α‐ketoglutarate—were detected. A broad metabolic differential diagnosis was considered, including primary or secondary mitochondrial disorders, urea cycle disorders, and hemophagocytic lymphohistiocytosis (HLH).

The patient was treated with a continuous intravenous infusion of 5% glucose solution. Electrolyte replacement therapy included potassium chloride and 3% hypertonic sodium chloride, administered over two days. Supportive medications included intravenous omeprazole and vitamin K once daily. For pain management and sedation, fentanyl was administered as a continuous infusion for 24 h.

Two days after her admission to the emergency department, the patient was transferred to the University Medical Center Hamburg‐Eppendorf in Germany for further management and potential liver transplantation, as this procedure is not available in Latvia. Due to the presence of pleural effusion, she was treated with antidiuretic medication. A liver biopsy was deemed unnecessary as her condition began to improve rapidly. Liver enzyme levels progressively declined, and she was discharged in stable condition 10 days after her initial presentation. Upon her return to Latvia, all laboratory results were within normal reference ranges and her liver size gradually normalized.

Metabolic disorder gene panel analysis from exome sequencing (with CNV calling) identified two variants in the *PCK1* gene (NM_002591.3): c.925G>A p.(Gly309Arg) and c.1833_1834del p.(Glu611AspfsTer16).

The first variant, c.925G>A p.(Gly309Arg), has been reported as a common pathogenic missense variant in the ClinVar database (Variation ID: 338886) and literature.

The second variant, c.1833_1834del p.(Glu611AspfsTer16), has not been previously reported either in population or clinical databases or in the literature. The variant is located in the last exon of the gene, so it is predicted to escape nonsense‐mediated degradation, causing a frameshift of the last 12 amino acids that participate in forming the structure of the PCK enzyme, replacing them with 16 different amino acids, likely disrupting the domain's (local) structure. The variants were confirmed to be in a compound heterozygous state via segregation analysis. Therefore, on the basis of the available information, the variant was classified as likely pathogenic.

Genetic testing of family members showed that the patient's 12‐year‐old sister carries the same *PCK1* gene variants but remains clinically asymptomatic. However, abdominal ultrasonography demonstrated a normal‐sized liver with mildly increased parenchymal echogenicity. Her medical history includes hospitalization for pneumonia at 2 months of age, during which she had normal blood glucose and liver enzyme levels. At age 3, she was two standard deviations below the average weight for her age and was evaluated for abdominal pain, nausea, poor appetite, and episodes of vomiting. She underwent fibrogastroscopy, which revealed mild, nonactive superficial duodenitis without atrophy. No other abnormalities were identified.

## 3 Discussion

Here, we report on two sisters, aged 8 and 12 years, who represent the first documented patients of PEPCK‐C deficiency in Latvia, including the first reported patient presenting with severe acute liver failure. In a systematic review of 32 individuals with PEPCK‐C deficiency, three patients were reported to have decreased liver function resembling acute liver failure during episodes of hypoglycemia; however, none of them met the PALF criteria [2, 7]. To our knowledge, this is the first documented patient of PEPCK‐C deficiency presenting with acute liver failure that fulfills PALF criteria, and in this patient, the condition was reversible.

Biochemically, the patient had markedly high glutamine levels in the absence of hyperammonemia. As Goetz et al. illustrate during ketotic state, dysfunction of PEPCK‐C leads to an accumulation of TCA cycle metabolites such as malate, fumarate, and alpha‐ketoglutarate. Secondary to alpha‐ketoglutarate, glutamate, and finally glutamine accumulate. Dysfunction of gluconeogenesis causes hyperlactatemia and hypoglycemia, which can be hyperketotic or possibly due to secondary inhibition of fatty acid beta oxidation, hypoketotic [2] The combination of hypoglycemia, elevated glutamine, and normal ammonia levels may serve as biochemical markers suggestive of PEPCK‐C deficiency in cases of potentially reversible acute liver failure.

In addition, the patient had acute kidney injury that has not been reported. It is difficult to determine whether kidney injury was caused by infection and contributes to disease manifestation or is a symptom of PEPCK‐C deficiency, but the *PCK1* gene is known to be expressed mainly in the liver and kidney [5].

The initial hypoglycemic episode in patients typically occurs during the neonatal period; however, the mean age at first clinical manifestation is 2.6 years (range, 0.5–13 years) [2]. In our proband, acute liver failure with hypoglycemia presented as the first manifestation of disease at 8 years of age, representing an atypical and delayed onset. Notably, her sister remains asymptomatic at 12 years of age. The most common triggers for the occurrence of a hypoglycemic episode reported were long periods of fasting or febrile infections with reduced food intake [2].

Potential triggers in this patient may have included infection and fasting, although the patient had experienced both previously without any manifestation of PEPCK‐C deficiency symptoms. A distinguishing factor in this episode was the amount of sucrose ingested immediately prior to symptom onset, accompanied by elevated urinary sucrose excretion. In a 2018 study, Latorre‐Muro et al. described the dynamic acetylation of *PCK1*, which interconverts the enzyme between gluconeogenic and anaplerotic activities, where with high energy input, Lys91 acetylation destabilizes the active site of *PCK1*, favors the reverse reaction, and promotes anaplerotic activity [8]. Therefore, it is hypothesized that the high energy load from sucrose ingestion prior to symptom onset contributed to enzyme destabilization, in combination with other factors.

Treatment of PEPCK‐C deficiency involves the avoidance of fasting and supplementation with additional carbohydrates during exercise, illness or other periods when the body needs additional sources of energy [9]. The PEPCK‐C enzyme is activated during periods of metabolic stress like fasting, intercurrent infections, trauma, or surgery, which explains the variation between reported cases in the age of onset that is dependent on metabolic stress events and on the genetic modifiers in other stress response pathways. That may also explain the asymptomatic state while patients are well. Taking early precautions to prevent metabolic stress and control its triggers would positively impact the patients' clinical state and eliminate the occurrence of irreversible cellular dysfunction in vital organs (i.e., brain and liver) [4]. Since there are no established guidelines for follow‐up, we conduct medical evaluations biannually and recommend avoidance of fasting and known metabolic triggers. Both sisters are actively engaged in gymnastics and are under close clinical observation. Additionally, given the potential risk of *PCK1* destabilization under high‐glucose conditions, they are advised to moderate their sugar intake and avoid consuming large quantities of rapidly absorbed sugars.

Evaluating the patients described in the literature alongside our clinical experience, we see that the symptoms have a wide range and are often self‐limiting. Different phenotypes, even among family members, create a risk that the true prevalence of PEPCK‐C deficiency is underestimated. This may lead to further risks for their siblings and offspring, who could potentially have more severe forms. In our opinion, it would be important to recommend NGS (Next‐Generation Sequencing) for everyone with altered glutamine and TCA cycle metabolites in the amino acid profile. However, regarding this patient, the NGS results were received late and the patient had already begun to improve. This could have led to premature decisions in the context of reversible liver failure, so using rapid exome sequencing would have been a better option.

On the other hand, unnecessary diagnostics for asymptomatic individuals cause stress for the family and an unnecessary burden on medical staff, since the natural course of the disease, monitoring protocols, and treatment are not clearly established.

In conclusion, we report a case of PEPCK‐C deficiency with severe reversible acute liver and kidney failure as the first clinical manifestation. We suggest that the combination of hypoglycemia, elevated glutamine, and normal ammonia levels could be indicative of PEPCK‐C deficiency. The previously unreported *PCK1* gene variant c.1833_1834del p.(Glu611AspfsTer16) should be classified as likely pathogenic.

## Author Contributions

I.G., D.R., and A.L. carried out the genetic diagnostics. I.P., M.A., and L.V. were responsible for patient's treatment and follow‐up. S.S.‐J. was responsible for the treatment carried out in Germany. M.A. supervised and planned the writing process of the article. L.V. was responsible for conducting and reporting the work described in the article. All authors approved the submitted version.

## Funding

The authors have nothing to report.

## Ethics Statement

The authors have nothing to report.

## Consent

All procedures followed were in accordance with the ethical standards of the responsible committee on human experimentation (institutional and national) and with the Helsinki Declaration of 1975, as revised in 2000 (5). Informed consent was obtained from all patients for being included in the study. Written informed consent was obtained from patients' parents prior to submission of the case report.

## Conflicts of Interest

The authors declare no conflicts of interest.

## Data Availability

The original contributions presented in the study are included in the article. Further inquiries can be directed to the corresponding author.
